# Genetically-Encoded Yellow Fluorescent cAMP Indicator with an Expanded Dynamic Range for Dual-Color Imaging

**DOI:** 10.1371/journal.pone.0100252

**Published:** 2014-06-24

**Authors:** Haruki Odaka, Satoshi Arai, Takafumi Inoue, Tetsuya Kitaguchi

**Affiliations:** 1 Cell Signaling Group, Waseda Bioscience Research Institute in Singapore (WABIOS), Waseda University, Singapore, Singapore; 2 Department of Life Science and Medical Bioscience, School of Advanced Science and Engineering, Waseda University, Shinjuku-ku, Tokyo, Japan; National Institute of Genetics, Japan

## Abstract

Cyclic AMP is a ubiquitous second messenger, which mediates many cellular responses mainly initiated by activation of cell surface receptors. Various Förster resonance energy transfer-based ratiometric cAMP indicators have been created for monitoring the spatial and temporal dynamics of cAMP at the single-cell level. However, single fluorescent protein-based cAMP indicators have been poorly developed, with improvement required for dynamic range and brightness. Based on our previous yellow fluorescent protein-based cAMP indicator, Flamindo, we developed an improved yellow fluorescent cAMP indicator named Flamindo2. Flamindo2 has a 2-fold expanded dynamic range and 8-fold increased brightness compared with Flamindo by optimization of linker peptides in the vicinity of the chromophore. We found that fluorescence intensity of Flamindo2 was decreased to 25% in response to cAMP. Live-cell cAMP imaging of the cytosol and nucleus in COS7 cells using Flamindo2 and nlsFlamindo2, respectively, showed that forskolin elevated cAMP levels in each compartment with different kinetics. Furthermore, dual-color imaging of cAMP and Ca^2+^ with Flamindo2 and a red fluorescent Ca^2+^ indicator, R-GECO, showed that cAMP and Ca^2+^ elevation were induced by noradrenaline in single HeLa cells. Our study shows that Flamindo2, which is feasible for multi-color imaging with other intracellular signaling molecules, is useful and is an alternative tool for live-cell imaging of intracellular cAMP dynamics.

## Introduction

Cyclic adenosine 3′,5′-monophosphate (cAMP) is an important intracellular second messenger, which mediates many cellular responses mainly initiated by the binding of hormones or neurotransmitters to cell surface receptors [1]. A variety of hormones or neurotransmitters, such as adrenaline, dopamine, and prostaglandins, stimulate specific G protein-coupled receptors and activate or suppress adenylate cyclase (AC), leading to an increase or decrease in cAMP. Cyclic AMP directly binds and activates at least three effectors, protein kinase A (PKA), exchange protein directly activated by cAMP (Epac), and cyclic nucleotide-gated channels, and mediates various cellular functions via distinct pathways [2]. Because some cAMP effector proteins are localized only in specific subcellular components, spatial and temporal cAMP dynamics are crucial for regulation of various cellular functions [3].

For investigation of spatial and temporal dynamics of intracellular messenger molecules, such as Ca^2+^, cAMP and cGMP, two types of genetically-encoded fluorescent indicators have been developed. These indicators are Förster resonance energy transfer (FRET)-based ratiometric indicators and single fluorescent protein (FP)-based intensiometric indicators. FRET-based ratiometric indicators enable monitoring of dynamics of intracellular molecules by a ratio of fluorescence intensity change at two different wavelengths. FP-based intensiometric indicators enable monitoring of dynamics of intracellular molecules by a change in fluorescence intensity of a single wavelength. To monitor cAMP dynamics in cells, several FRET-based cAMP sensors have been generated [4]–[9]. The FRET-based cAMP indicators contain a cAMP binding domain in the effector molecules (i.e., PKA, Epac, and cyclic nucleotide-gated channels). These are tagged with a pair of FPs, such as cyan FP and yellow FP, for detecting a change in FRET signal induced by binding of cAMP to the domains. Although live-cell imaging using FRET-based cAMP indicators can visualize spatial and temporal dynamics of cAMP, two different emission wavelengths are required for measurement. This limits available wavelengths for multi-color imaging in combination with other signaling molecule indicators. A single FP-based intensiometric indicator is a potential alternative for multi-color imaging because it only requires a single wavelength measurement. Intensiometric indicators usually consist of a binding domain of intracellular signaling molecules inserted in the vicinity of the chromophore of a fluorescent protein. The dynamic conformational changes induced by binding to target molecules result in changes in fluorescence intensity. Several single FP-based indicators for Ca^2+^, cGMP, H_2_O_2_, protein phosphorylation, the ATP:ADP ratio, and glutamate have been reported, whereas single FP-based cAMP indicators are poorly established (Table 1) [10]–[20]. We previously showed that the single FP-based intensiometric cAMP indicator, Flamindo (fluorescent cAMP indicator), can successfully monitor cAMP dynamics in the cytoplasm and subplasma membrane [20]. However, Flamindo has a small dynamic range and low fluorescence intensity.

**Table 1:** Comparison of single FP-based indicators.

Name	Detection	Ex/Em (nm)	Type	Dynamic range (fold)	Ref
G-CaMP	Ca^2+^	489/509	I	4.5	[10]
G-CaMP8	Ca^2+^	498*/508*	I	38*	[11]
Flash-pericam	Ca^2+^	490*/514	I	8	[12]
G-GECO1	Ca^2+^	496*/512	I	25	[13]
R-GECO1	Ca^2+^	561*/589	I	16	[13]
FlincGβ	cGMP	480/510	I	2.1	[14]
HyPer	H_2_O_2_	420, 500/516	R	3.3	[15]
cyan-sinphos	phosphorylation	430/480*	I	1.1*	[16]
Perceval	ATP:ADP ratio	405, 490/530	R	1.8*	[17]
PercevalHR	ATP:ADP ratio	420, 500/530*	R	8	[18]
iGluSnFR	glutamate	485*/515*	I	4.5	[19]
Flamindo	cAMP	506/521	I	2	[20]
Flamindo2	cAMP	504/523	I	4	

I, Intensiometric; R, Ratiometric.

*Estimated from graphs or descriptions.

We report here an improved yellow fluorescent cAMP indicator named Flamindo2. In Flamindo2, linker peptides in the vicinity of the chromophore in Flamindo are optimized, which has drastically expanded the dynamic range and increased brightness. Because genetically-encoded single FP-based indictors are suitable for multi-color imaging and targeting organelles, Flamindo2 is promising for monitoring intracellular cAMP dynamics with a high spatio-temporal resolution.

## Materials and Methods

### 2.1. Chemicals

Cyclic AMP, 8-bromo-cAMP (8-Br-cAMP), and forskolin were purchased from Calbiochem. Cyclic GMP, 3-isobutyl-1-methylxanthine (IBMX), and noradrenaline were purchased from Sigma-Aldrich. Bicarbonate was purchased from Life technologies.

### 2.2. Plasmid construction

Flamindo consists of an yellow FP variant, Citrine, fused with a cAMP binding domain of mEPAC1 (GenBank NP_001171281, 199–358 aa) in the vicinity of its chromophore. To improve the performance of Flamindo, we created many candidates by addition or deletion of amino acids in the N-terminus or C-terminus of the cAMP binding domain by PCR (Fig. S1). A mutant indicator with the largest dynamic range was named Flamindo2, and cloned into the BamHI/HindIII sites of pcDNA3.1 (–) and pRSET_B_ vectors for expression in mammalian cells and bacterial cells, respectively. For nuclear localization of Flamindo2, the nuclear localization signal sequence (MPKKKRKVEDVDP) was fused to the N-terminus of Flamindo2 and cloned into pcDNA3.1 (–). R-GECO, a red fluorescent genetically-encoded Ca^2+^ indicator, was generated by DNA synthesis (Integrated DNA Technologies) and inserted into the BamHI/EcoRI site of the pcDNA3 vector for mammalian expression [13].

### 2.3. Protein expression and *in vitro* spectroscopy

Extraction and purification of Flamindo/Flamindo2 protein and *in vitro* spectroscopy were performed as previously reported [20]. Briefly, a Flamindo/Flamindo2-containing pRSET_B_ vector was introduced into *Escherichia coli* JM109 (DE3) cells and cells were cultured at 20°C for 4 days. After 4 days of culture, cells were harvested by centrifugation, lysed by three freeze-thaw cycles, and sonicated with lysozyme. After centrifugation of the lysate, His-tagged Flamindo/Flamindo2 protein was purified from supernatants using a Ni-NTA agarose column (Qiagen) followed by a PD-10 gel-filtration column (GE Healthcare). The purified protein was finally eluted in Hepes buffer (150 mM KCl and 50 mM Hepes-KOH [pH 7.4]). The concentration of purified Flamindo/Flamindo2 protein was measured by the Bradford protein assay using Bio-Rad Protein Assay (Bio-Rad). Bovine serum albumin was used as a standard. Absorption and fluorescence spectra of purified Flamindo/Flamindo2 protein were measured using a UV-670 UV-Vis spectrophotometer (Jasco) and F-2700 fluorescence spectrophotometer (Hitachi), respectively.

### 2.4. Cell culture and transfection

COS7 (ATCC CRL-1651) and HeLa (ATCC CCL-2) cells were cultured in Dulbecco's modified Eagle's medium (DMEM) containing 10% fetal bovine serum and penicillin/streptomycin on a 100-mm dish at 37°C under 5% CO_2_.

For live-cell imaging, COS7 cells were plated onto glass coverslips in a 35-mm dish, and cells on the glass were transfected with 0.2 µg of Flamindo2 or nlsFlamindo2 using 0.8 µl FuGENE HD Transfection Reagent (Promega) and 10 µl Opti-MEM (Life Technologies Corporation). For dual-color live-cell imaging, HeLa cells were plated on glass coverslips in a 35-mm dish, and were also transfected with 0.1 µg of Flamindo2 and 0.1 µg R-GECO using 0.8 µl FuGENE HD and 10 µl Opti-MEM. After transfection, cells were incubated at 28°C for 2 to 4 days until fluorescence imaging. For bicarbonate stimulation, cells were incubated in phenol red and bicarbonate free-DMEM for 3 hours before fluorescence imaging.

### 2.5. Fluorescence imaging

Fluorescence imaging of Flamindo2/nlsFlamindo2 was performed in phenol red free-DMEM or phenol red and bicarbonate free-DMEM under an Olympus IX 81 inverted microscope with a cooled CCD camera (Cool SNAP HQ^2^, Photometrics). A UPlanFL N 40×1.30 numerical aperture (NA) and oil-immersion objective lens (Olympus) was used. Images were acquired and analyzed with MetaFluor software (Molecular Devices). A 488–512 nm excitation filter, 520 nm dichroic mirror, and 528.5–555.5 nm emission filter (Semrock) were used for measurement of a single wavelength of Flamindo2. For dual-color imaging of Flamindo2 and R-GECO, two excitation filters (460–480 nm filter for Flamindo2 and 535–555 nm filter for R-GECO), a dual-band dichroic mirror (493/574 nm), and two emission filters (495–540 nm filter for Flamindo2 and 570–625 nm filter for R-GECO) (Olympus) were alternated by using an HF110 high speed filter wheel (Prior Scientific). Images were acquired every 5 s.

## Results

### 3.1. Improvement of a yellow fluorescent indicator for cAMP

**Figure 1:**
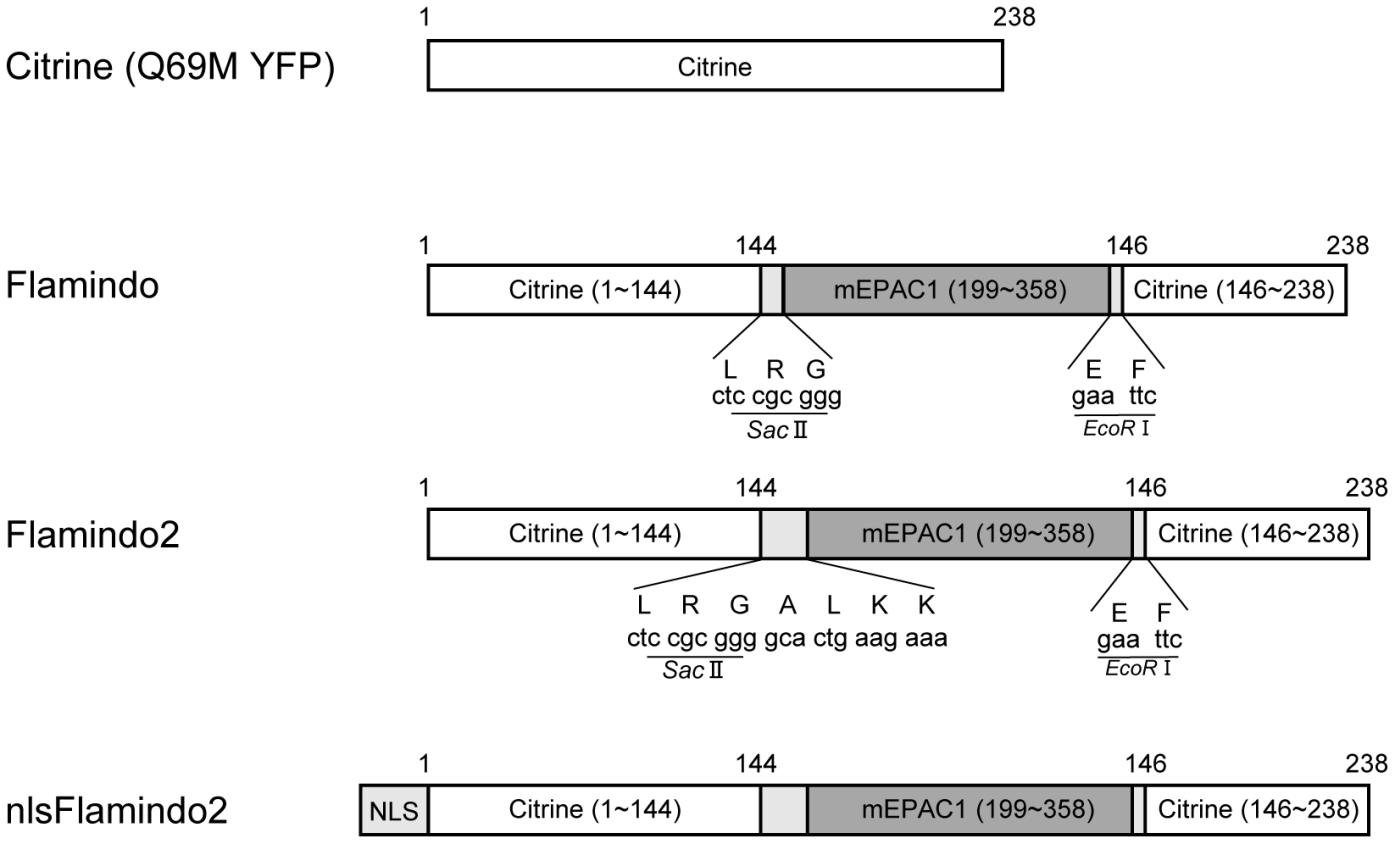
Schematic representation of the domain structure of Flamindo2. Citrine is a mutant of yellow fluorescent protein (YFP). cDNAs for yellow fluorescent cAMP indicators, Flamindo and Flamindo2, were created by insertion of DNA fragments encoding the cAMP-binding domain of mEPAC1 (199–358) and N- and C- terminus linker peptides including restriction sites. Nucleus-targeted Flamindo2 (nlsFlamindo2) was created by fusion of NLS (nuclear localization signal; MPKKKRKVEDVDP) to the N-terminus of Flamindo2.

The yellow fluorescent indicator for cAMP, Flamindo, is composed of an yellow fluorescent protein variant, Citrine, fused with a cAMP binding domain of mouse Epac1 (exchange protein directly activated by cAMP) in the vicinity of its chromophore [20]. Flamindo converts the conformational change induced by cAMP binding into changes in fluorescence intensity. Previous studies have shown that alterations in the length and/or type of amino acids in the linker sequence between the binding domain of the analyte and the fluorescent protein improve the dynamic range of indicators upon analyte binding [10], [12]. Therefore, we previously created four Flamindo variants by deletion or addition of three amino acids in the N- or C-terminus of the cAMP binding domain for expansion of the dynamic range of Flamindo [20]. In the current study, we chose a linker amino acid sequence based on the N-terminus of the NZ leucine zipper. This choice was based on the assumption that a strong alpha helix structure, such as an NZ leucine zipper, may affect the microenvironment in the vicinity of the chromophore, which leads to improvements on brightness and dynamic range [21]. Although we found an insignificant improvement in Flamindo variants in our previous study, one variant with insertion of three amino acids to the N-terminus of the cAMP binding domain displayed a similar dynamic range as Flamindo [20]. Therefore, we focused on insertion of amino acids to the N-terminus of the cAMP binding domain. Three additional variants with various lengths of amino acids (6, 9, and 12 amino acids) between Leu-Arg and Gly showed little improvement in the dynamic range (Fig. S1). In another five additional variants with different lengths of amino acid linkers downstream of the Leu-Arg-Gly sequence, one variant with an ALKK linker insertion displayed the largest change in fluorescence intensity upon addition of cAMP (Fig. S1). This was named Flamindo2 and subjected to further characterization (Fig. 1).

### 3.2. Spectral characteristics of Flamindo2

**Figure 2:**
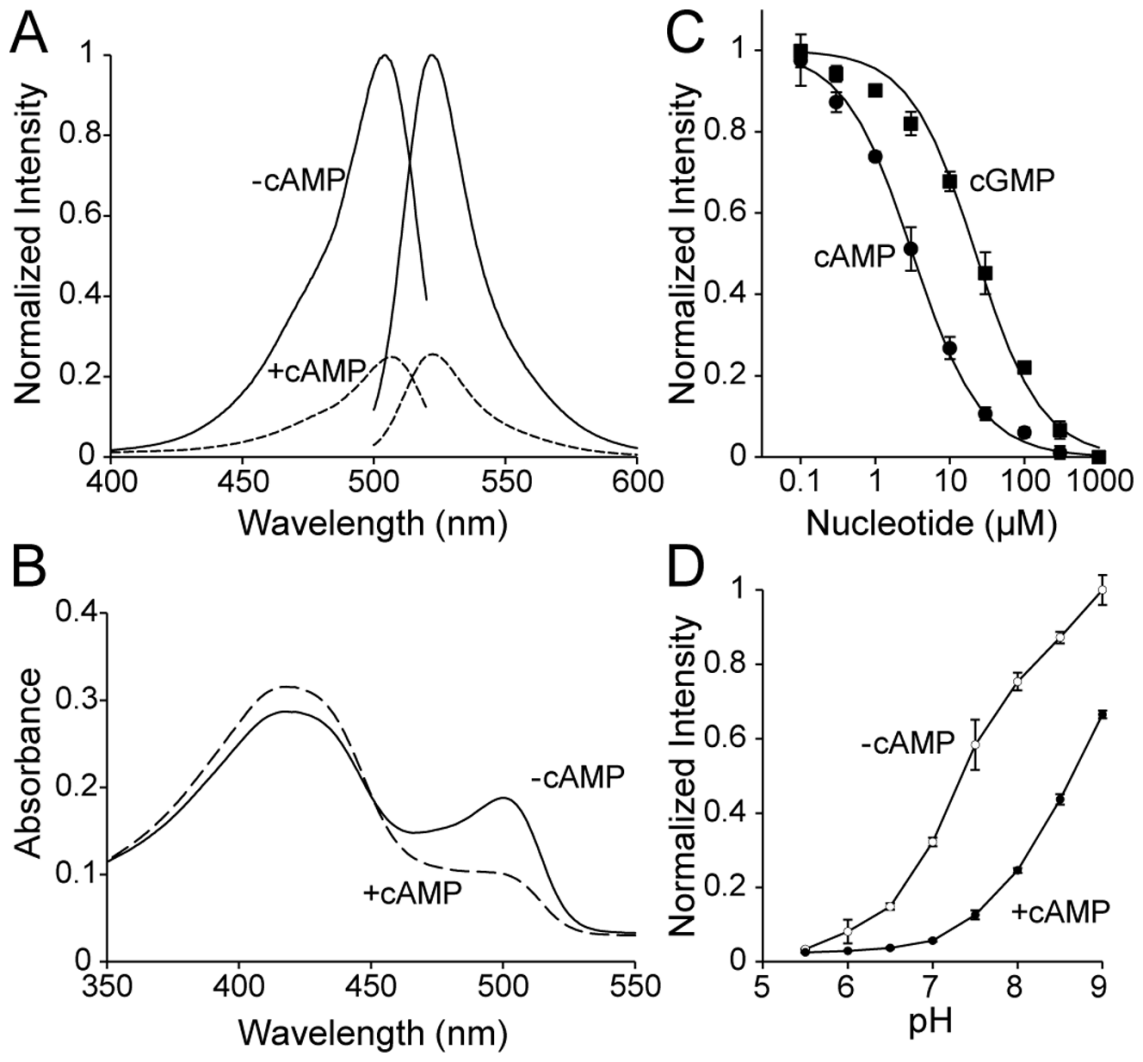
Spectral characterization of Flamindo2. (A) Fluorescence spectra of Flamindo2 protein. Excitation and emission spectra were measured in the presence (dotted lines, +cAMP) or absence (solid lines, -cAMP) of 1 mM cAMP. Each fluorescence intensity (FI) was normalized to the peak of FI in the absence of cAMP. The representative excitation/emission spectra data from three independent experiments was shown in the graph. (B) Absorbance spectra of Flamindo2 protein in the presence (dotted line, +cAMP) or absence (solid line, -cAMP) of 1 mM cAMP. The representative absorption spectra data from three independent experiments was shown in the graph. (C) Titration curves of cAMP and cGMP. The peak of FI at each concentration of cAMP (closed circles) or cGMP (closed squares) was normalized to the peak of FI in the absence of cAMP or cGMP. The results are mean ± SD (n = 3) (D) Titration curves against pH. The peak of FI at each pH in the presence (closed circles) or absence (open circles) of 1 mM cAMP was normalized to the peak of FI at pH 9.0 in the absence of cAMP. The results are mean ± SD (n = 3).

To investigate spectral properties of Flamindo2, we first measured the fluorescence excitation and emission spectra of purified Flamindo2 protein. These spectra showed an excitation peak at 504vnm and an emission peak at 523 nm. A saturating dose of cAMP (1 mM) decreased the fluorescence intensity by 75% (4-fold change) (Fig. 2A). Because 1 mM cAMP decreased the fluorescence intensity of Flamindo by 50% (2-fold change) [20], the dynamic range of Flamindo2 was 2-fold larger than that of Flamindo (Fig. S2). Furthermore, the brightness of Flamindo2 was improved by 8-fold without spectral property changes (Fig. S2). The absorption spectra of 20 µM Flamindo2 protein showed a larger decrease at 500 nm and increase at 421 nm of the peak upon cAMP binding compared with Flamindo (Fig. 2B). Since the absorption peak at approximately 400 nm corresponds to the protonated form of the chromophore and the absorption peak at approximately 500 nm corresponds to the deprotonated form, cAMP binding to Flamindo2 increased the proportion of protonated chromophore, contributing to a decrease in fluorescence intensity. To examine the specificity of Flamindo2 for cAMP, we performed cAMP and cGMP titration of Flamindo2, and calculated the dissociation constant (K_d_) values (Fig. 2C). The Hill plots for cAMP and cGMP showed that the K_d_ values were 3.2 µM and 22 µM, respectively, which are close to those of Flamindo (cAMP: K_d_ = 2.1 µM, cGMP: K_d_ = 22 µM, Fig. S3). Hill coefficients for cAMP and cGMP were 0.95 and 0.98, respectively, suggesting that binding of cAMP or cGMP to Flamindo2 is not involved in any allosteric reactions. In addition, the pH sensitivity of fluorescence intensity of Flamindo2 was similar to that of many single wavelength indicators, including Flamindo (i.e., G-CaMP, pericam, and GECO) (Fig. 2D) [10], [12], [13]. This pH sensitivity reflects pH-dependent protonation/deprotonation changes of chromophores [22]. These results indicate that Flamindo2 has improved usability with an expanded dynamic range and greater brightness than Flamindo.

### 3.3 Live-cell imaging of cAMP dynamics in Flamindo2-expressing COS7 cells

**Figure 3:**
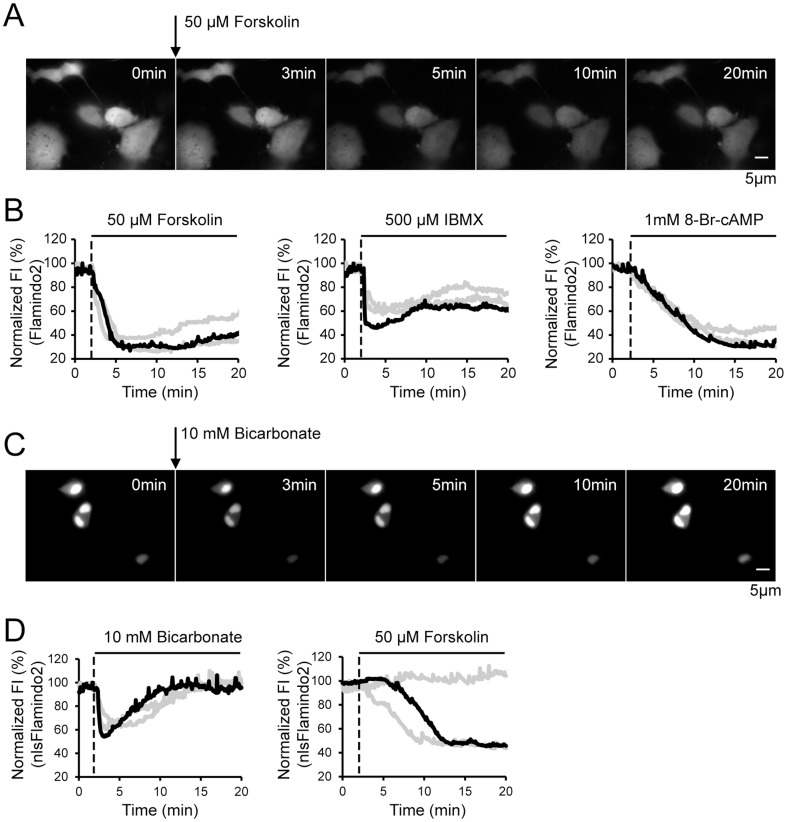
Live-cell imaging of cAMP in Flamindo2/nlsFlamindo2-expressing COS7 cells. (A) Representative images showing changes in fluorescence intensity (FI) by 50 µM forskolin application in Flamindo2-expressing COS7 cells. (B) Time course of a normalized decrease in FI induced by reagents, which increase intracellular cAMP levels. Each reagent was applied at 2 min (dotted line). Three traces (black and gray lines) from single cells in three independent experiments are shown in all of the graphs. The black traces indicate representative data. (C) Representative images showing changes in FI by 10 mM bicarbonate application in nlsFlamindo2-expressing COS7 cells. (D) Different effects of bicarbonate and forskolin on cAMP dynamics in the nucleus. Bicarbonate or forskolin was applied at 2 min. Three traces (black and gray lines) from single cells in three independent experiments are shown in all of the graphs. The black trace indicates representative data.

To examine whether Flamindo2 functions in living cells, we transfected Flamindo2 DNA into COS7 cells. We then investigated the effects of forskolin, IBMX, and 8-Br cAMP, which increase intracellular cAMP levels via distinct pathways. Application of 50 µM forskolin, an activator of AC, caused a 70% decrease in fluorescence intensity within 3 min (Fig. 3A, B). Correspondingly, application of 500 µM IBMX, an inhibitor of phosphodiesterase, and 1 mM 8-Br cAMP, a membrane permeable cAMP analogue, caused 40–55% and 70% decrease in fluorescence intensity, respectively. These results indicate that Flamindo2 is suitable for monitoring cAMP dynamics in live cells.

### 3.4 Imaging of cAMP dynamics in the nucleus with nlsFlamindo2

Signaling molecules, as well as the enzymes producing them and their effectors, are localized in subcellular compartments, such as the cytoplasm, nucleus, and mitochondria [3], [23]. Therefore, subcellular resolution in monitoring dynamics of signaling molecules is essential for understanding the physiological functions of cells. Genetically-encoded fluorescence indicators are widely used to investigate molecular dynamics in subcellular compartments because they are easily localized to subcellular compartments by fusing with localization signal sequences. Bicarbonate and forskolin stimulate different types of AC localized in the nucleus and plasma membrane, respectively [24], [25]. Therefore, we tested these stimulants on cAMP dynamics in the nucleus by using a nuclear localization signal fused with Flamindo2, namely nlsFlamindo2. Expression of nlsFlamindo2 was mainly observed in nuclei in COS7 cells (Fig. 3C). Bicarbonate treatment caused a rapid decrease in fluorescence intensity in nuclei followed by a return to basal levels. However, forskolin treatment caused a delayed and slow decrease, or no detectable change in fluorescence intensity (Fig. 3D). This delay in forskolin-induced increase of cAMP in the nucleus may be attributed to diffusion of cAMP from the cytosol to the nucleus. These results suggest that different localizations of AC result in non-uniform cAMP dynamics in cells, leading to complex spatio-temporal regulation patterns of cAMP effector molecules.

### 3.5 Dual-color imaging of cAMP and Ca^2+^ in HeLa cells

**Figure 4:**
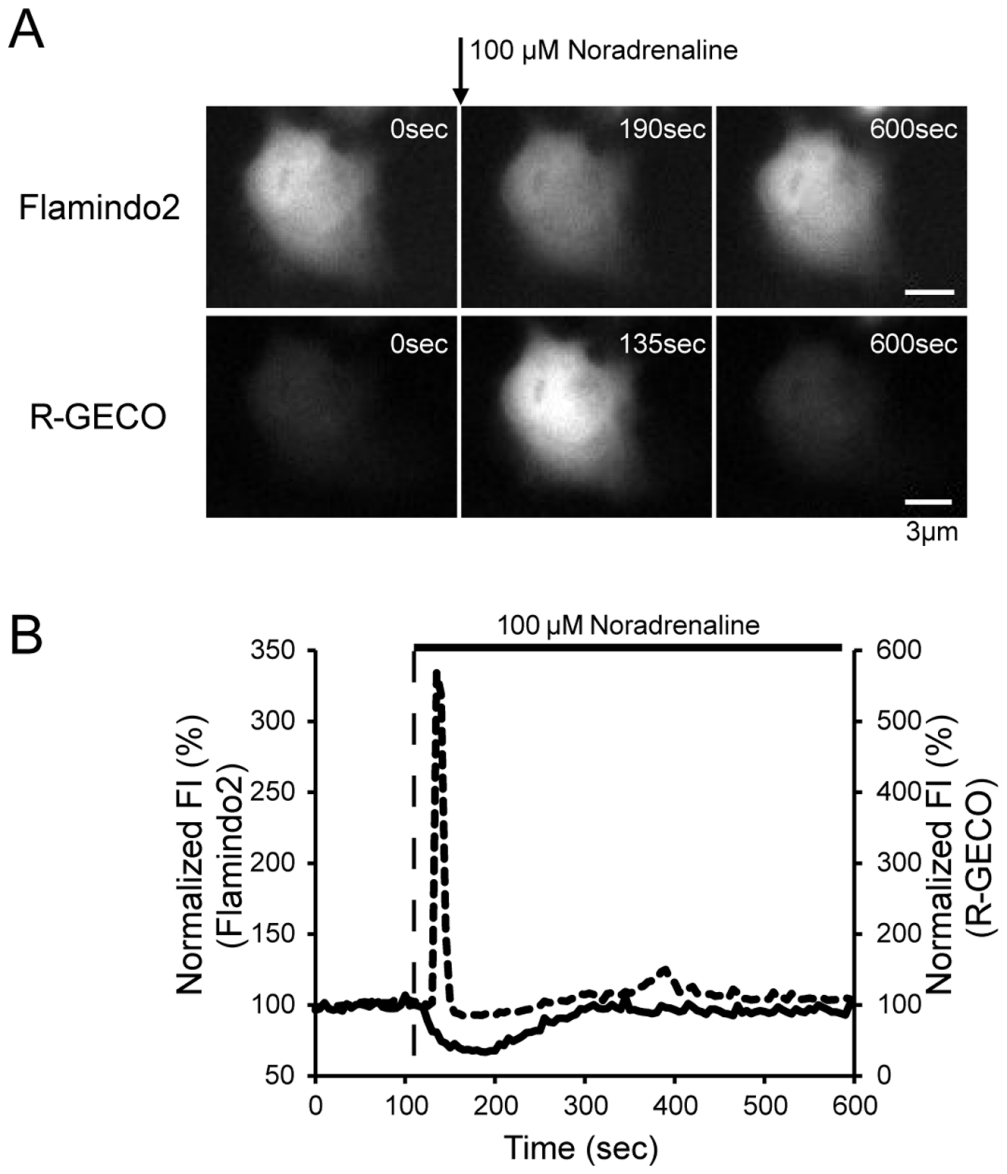
Dual-color imaging of cAMP and Ca^2+^ in Flamindo2 and R-GECO co-expressing HeLa cells. (A) Images of changes in fluorescence of Flamindo2 and R-GECO induced by 100 µM noradrenaline. (B) Time course of changes in cAMP (solid line) and Ca^2+^ (dotted line) induced by 100 µM noradrenaline. Noradrenaline was applied at 120 s. Representative trace is shown in the graph.

Both cAMP and Ca^2+^ are important second messengers, which modulate numerous cellular functions, and various hormones and neurotransmitters regulate intracellular cAMP and/or Ca^2+^ concentrations via G protein coupled receptors [2]. To precisely understand the cellular functions involved in cAMP and Ca^2+^ signaling, monitoring the dynamics of both molecules in the same cell is essential. To achieve this, we co-transfected HeLa cells with Flamindo2 and R-GECO, the latter of which is a genetically-encoded red fluorescent Ca^2+^ indicator (Fig. 4A), and performed dual-color imaging. Application of 100 µM noradrenaline caused a transient increase in R-GECO fluorescence intensity, and gradual decrease and recovery of Flamindo2 fluorescence intensity (Fig. 4B). Time to peak fluorescence intensity of R-GECO by noradrenaline application was significantly shorter than that of Flamindo2 (Fig. S4B). This finding is consistent with previous reports that noradrenaline increases intracellular cAMP and Ca^2+^ levels in HeLa cells [26], [27]. This result shows that Flamindo2 is suitable for dual-color imaging with other red fluorescent indicators.

## Discussion

In the present study, we developed improved yellow fluorescent cAMP indicator, named Flamindo2. Flamindo2 shows 2-fold and 8-fold increases in dynamic range and brightness, respectively, compared with Flamindo. However, Flamindo2 still suffers from the pH sensitivity, as well as Flamindo does. The pH sensitivity found in Flamindo2 (Fig. 2D) is commonly observed in single fluorescent indicators because pH changes directly affect the protonation state of chromophores. To rule out pH-dependent changes in fluorescence intensity upon stimulation, we have shown that stimuli did not change fluorescence intensity through changes in pH in the cell by using mutant Flamindo (Flamindo [R279E]) [20]. During dual-color imaging in HeLa cells, Flamindo2 and R-GECO did not show identical fluorescence intensity changes by noradrenaline treatment, suggesting that noradrenaline stimulation does not cause apparent pH changes in HeLa cells (Fig. 4B).

Activation of AC induces production of cAMP via catalyzing the conversion of ATP to cAMP [1]. In mammalian cells, nine 12-transmembrane ACs (tmACs) and one soluble AC (sAC) have been identified [25]. The tmACs are localized on the plasma membrane and regulated by cell surface receptors, while sAC is distributed in the cytosol and partly in nuclei where it modulates gene expression [28]. Transmembrane ACs and sAC are selectively activated by forskolin and bicarbonate, respectively [24], [25]. A previous study reported that the phosphorylation level of cAMP response element binding protein (CREB) localized in the nucleus is rapidly increased by bicarbonate treatment within 2 min, while forskolin activates CREB only after 5 min [24]. These different kinetics of CREB phosphorylation may reflect the distance between the plasma membrane and the nucleus. In our study using Flamindo2, bicarbonate treatment induced a rapid and acute increase in cAMP in the nucleus, while forskolin caused a delayed and slow, or no increase in cAMP in the nucleus. These consistent results indicate that organellar expression of Flamindo2 is promising for detecting cAMP dynamics in subcellular compartments.

To understand complex intracellular signaling transduction, dual-color monitoring of two signaling molecules is essential. We performed dual-color imaging of cAMP and Ca^2+^ in HeLa cells with noradrenaline stimulation. β-adrenergic receptors, which increase cAMP production via activating AC through Gsα, and the α1-adrenergic receptor, which increases intracellular Ca^2+^ levels via activating the phospholipase C/inositol 1, 4, 5-trisphosphate pathway through Gq, are expressed in HeLa cells [26], [27], [29]. Noradrenaline acts on the both receptors, leading to an increase in cAMP and Ca^2+^ through distinct pathways. In our study, although the onset time of an increase in cAMP and Ca^2+^ after noradrenaline application was nearly identical, the kinetics of cAMP and Ca^2+^ responses were clearly distinguishable (Fig. 4, Fig. S4). Because intracellular cAMP levels in the cell are mainly regulated by the balance of activities of AC and phosphodiesterase, cAMP kinetics are thought to be relatively slow. In contrast, increase and decrease of Ca^2+^ levels in the cell is generally rapid, because Ca^2+^ influx through Ca^2+^ channels determines the Ca^2+^ response [30]. Numerous hormones and neurotransmitters affect the dynamics of cAMP and Ca^2+^. Therefore, dual-color imaging with Flamindo2 and R-GECO will reveal new molecular interactions between cAMP and Ca^2+^ at the single-cell level.

In summary, we generated improved cAMP indicator, Flamindo2 and showed cAMP dynamics in live cells. We also succeeded in visualizing the spatial distribution of cAMP production with nlsFlamindo2 by two distinct stimuli. Furthermore, we also showed different kinetics of Ca^2+^ and cAMP dynamics evoked by noradrenaline treatment by dual-color imaging. Therefore, Flamindo2 is useful for visualizing subcellular localized cAMP dynamics and for dual-color imaging with another red fluorescent indicator. We believe that Flamindo2 will be a strong tool for the study of cAMP dynamics in live cells.

## Supporting Information

Figure S1 **Schematic representation of the domain structure of mutated Flamindo.** Cell lysates from mutated Flamindo-expressing JM109 (DE3) were subjected to fluorescence spectrophotometry with or without 1 mM cAMP. A “response” represents the normalized dynamic range of each mutated Flamindo compared with that of Flamindo. Each of the dynamic ranges was measured from crude cell lysate of protein-expressing JM109 (DE3) cells with or without 1 mM cAMP, and they were normalized by the dynamic range of Flamindo.
(TIF)

Figure S2 **Fluorescence spectra of Flamindo and Flamindo2.** (A) Excitation and emission spectra of Flamindo (1 µM, gray lines) and Flamindo2 (1 µM, black lines) were measured in the presence (dotted lines, +cAMP) or absence (solid lines, -cAMP) of 1 mM cAMP. Each fluorescence intensity (FI) was normalized to the peak of FI of Flamindo in the absence of cAMP. Flamindo2 data are the same as Figure 2A. The representative excitation/emission spectra from three independent experiments was shown in the graph. (B) Dynamic range (left) and brightness (right) of Flamindo and Flamindo2. Dynamic range was calculated by each emission peak of Flamindo/Flamindo2 with or without 1 mM cAMP. The brightness was evaluated by the emission peak of Flamindo/Flamindo2 normalized to the peak of Flamindo in the absence of cAMP. The results shown are mean ± SD (n = 3). ***P<0.001 (t-test).
(TIF)

Figure S3 **Dose-response curves of Flamindo and Flamindo2 for cAMP/cGMP.** The gray line represents FI of Flamindo, and the black line represents that of Flamindo2. The peak of FI at each concentration of cAMP (closed circles) or cGMP (closed squares) was normalized to the peak of FI in the absence of cAMP or cGMP. Flamindo2 curves are the same as Figure 2C. The results shown are mean ± SD (n = 3).
(TIF)

Figure S4 **Different kinetics of cAMP and Ca^2+^ responses.** (A) Time course of changes in FI of Flamindo2 (solid lines) and R-GECO (dotted lines) induced by 100 µM noradrenaline. Noradrenaline was applied at 120 s. Three traces (black and gray lines) from single cells in three independent experiments are shown in the graphs. The black traces indicate representative data and are the same as Figure 4B. (B) Time to peak fluorescence intensity of Flamindo2 and R-GECO by noradrenaline application. The time to peak is the time from noradrenaline application (120 s) until peak of FI for each indicator. The results shown are mean ± SD (n = 3). *P<0.05 (t-test).
(TIF)
