# Supplementary figures and images for: Genetically-Encoded Yellow Fluorescent cAMP Indicator with an Expanded Dynamic Range for Dual-Color Imaging
Source: PLoS One. 2014 Jun 24;9(6):e100252. doi: 10.1371/journal.pone.0100252 (PMC4069001; doi:10.1371/journal.pone.0100252)

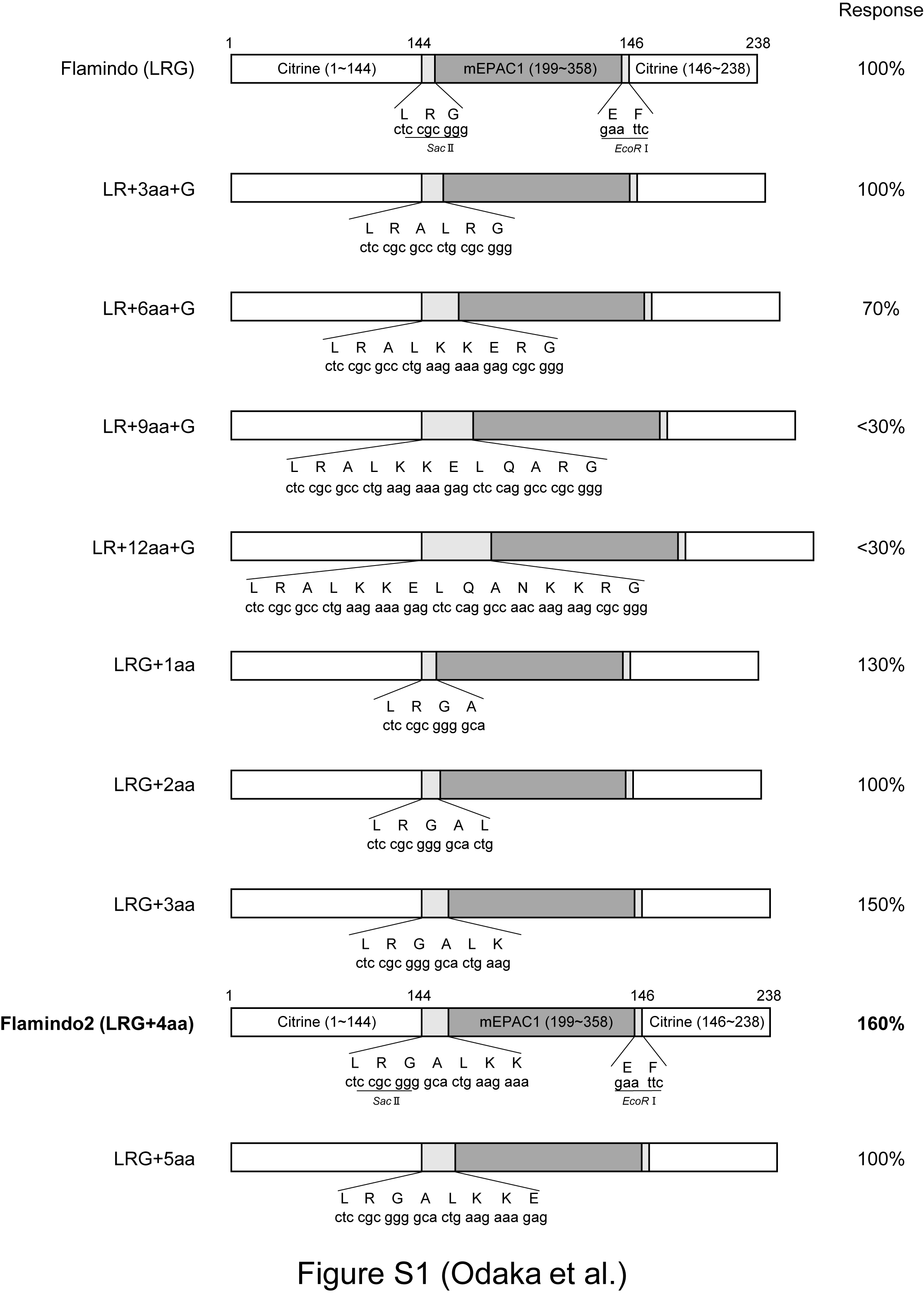

Supplement: Figure S1 — Schematic representation of the domain structure of mutated Flamindo. Cell lysates from mutated Flamindo-expressing JM109 (DE3) were subjected to fluorescence spectrophotometry with or without 1 mM cAMP. A “response” represents the normalized dynamic range of each mutated Flamindo compared with that of Flamindo. Each of the dynamic ranges was measured from crude cell lysate of protein-expressing JM109 (DE3) cells with or without 1 mM cAMP, and they were normalized by the dynamic range of Flamindo. (TIF) [file pone.0100252.s001.tif]

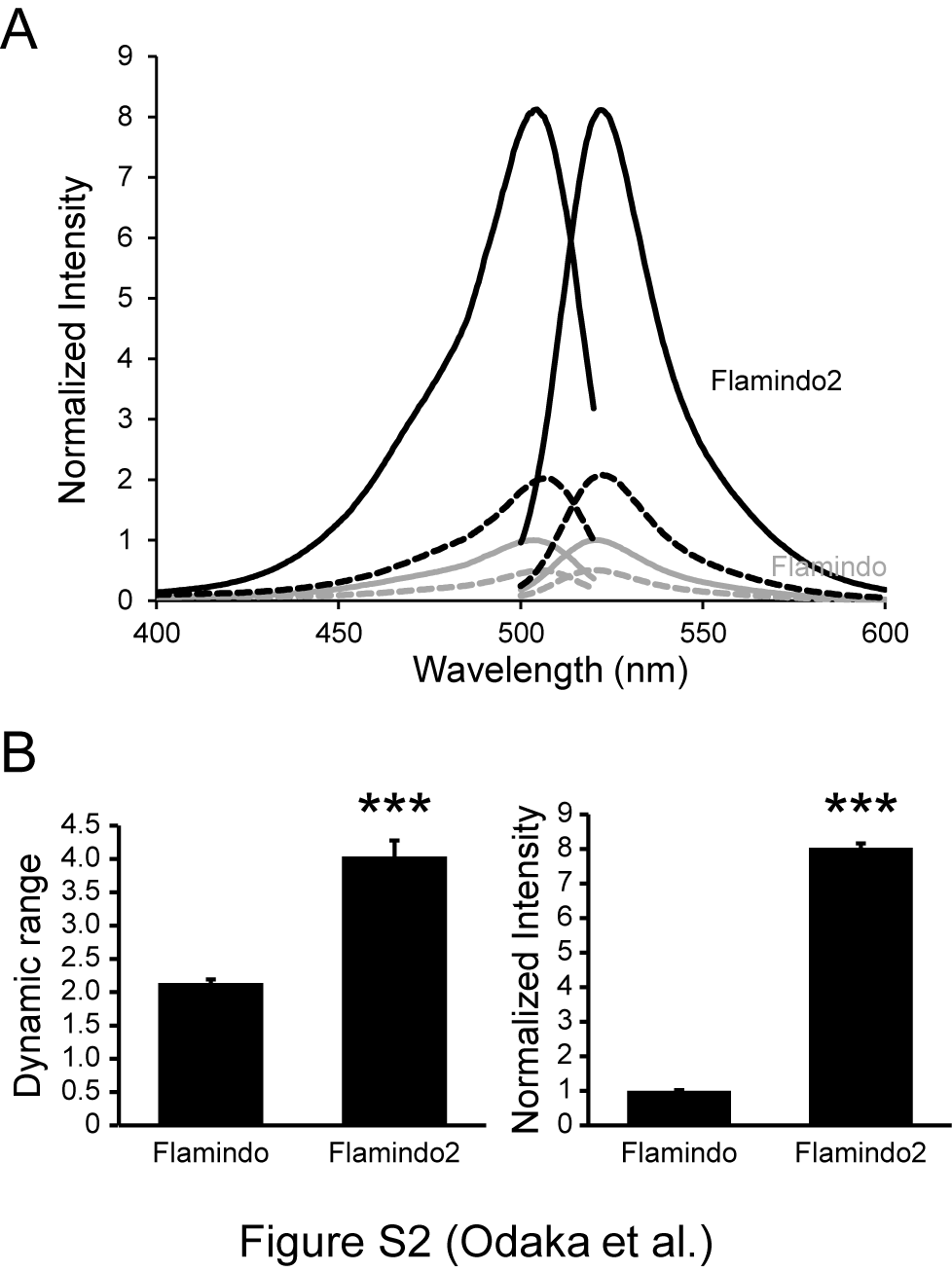

Supplement: Figure S2 — Fluorescence spectra of Flamindo and Flamindo2. (A) Excitation and emission spectra of Flamindo (1 µM, gray lines) and Flamindo2 (1 µM, black lines) were measured in the presence (dotted lines, +cAMP) or absence (solid lines, -cAMP) of 1 mM cAMP. Each fluorescence intensity (FI) was normalized to the peak of FI of Flamindo in the absence of cAMP. Flamindo2 data are the same as Figure 2A. The representative excitation/emission spectra from three independent experiments was shown in the graph. (B) Dynamic range (left) and brightness (right) of Flamindo and Flamindo2. Dynamic range was calculated by each emission peak of Flamindo/Flamindo2 with or without 1 mM cAMP. The brightness was evaluated by the emission peak of Flamindo/Flamindo2 normalized to the peak of Flamindo in the absence of cAMP. The results shown are mean ± SD (n = 3). ***P<0.001 (t-test). (TIF) [file pone.0100252.s002.tif]

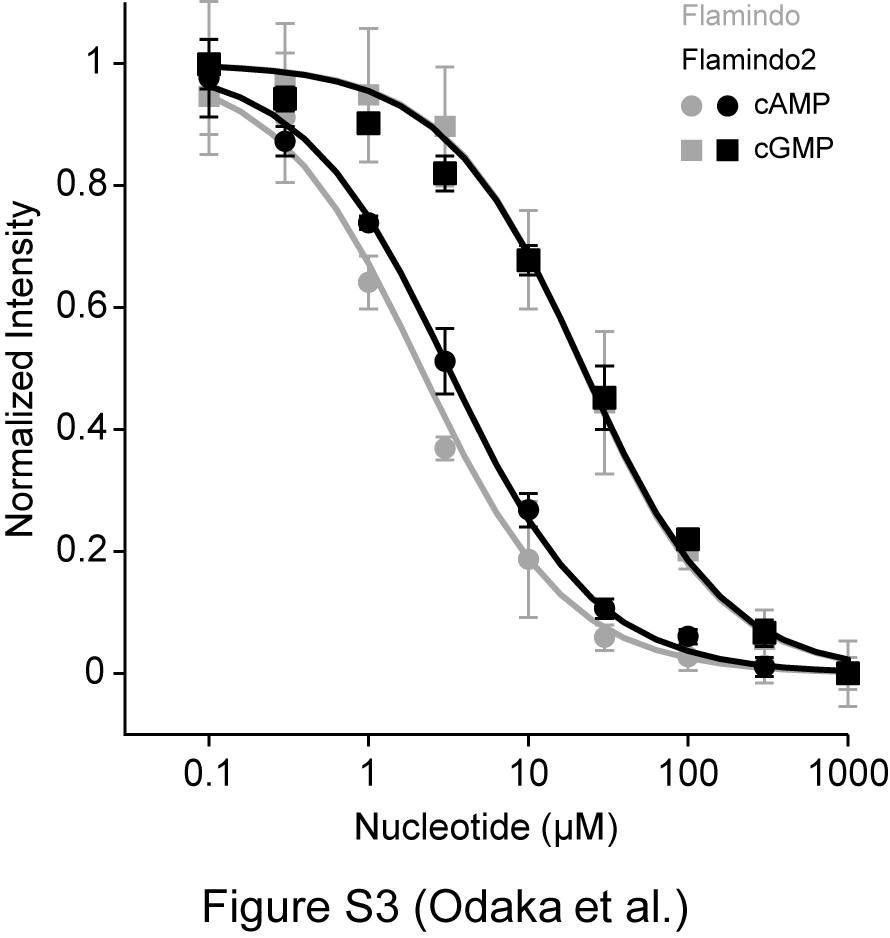

Supplement: Figure S3 — Dose-response curves of Flamindo and Flamindo2 for cAMP/cGMP. The gray line represents FI of Flamindo, and the black line represents that of Flamindo2. The peak of FI at each concentration of cAMP (closed circles) or cGMP (closed squares) was normalized to the peak of FI in the absence of cAMP or cGMP. Flamindo2 curves are the same as Figure 2C. The results shown are mean ± SD (n = 3). (TIF) [file pone.0100252.s003.tif]

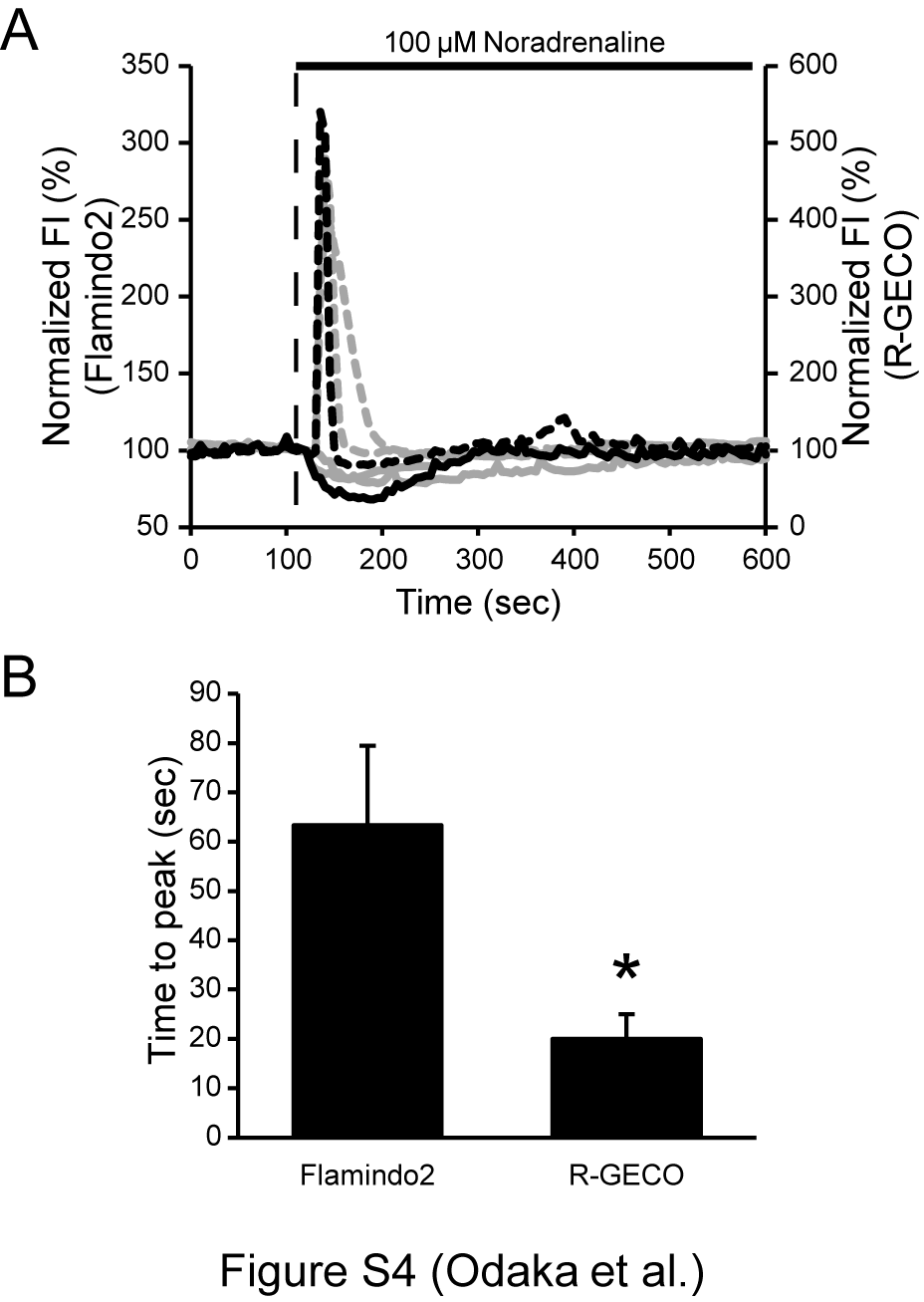

Supplement: Figure S4 — Different kinetics of cAMP and Ca2+ responses. (A) Time course of changes in FI of Flamindo2 (solid lines) and R-GECO (dotted lines) induced by 100 µM noradrenaline. Noradrenaline was applied at 120 s. Three traces (black and gray lines) from single cells in three independent experiments are shown in the graphs. The black traces indicate representative data and are the same as Figure 4B. (B) Time to peak fluorescence intensity of Flamindo2 and R-GECO by noradrenaline application. The time to peak is the time from noradrenaline application (120 s) until peak of FI for each indicator. The results shown are mean ± SD (n = 3). *P<0.05 (t-test). (TIF) [file pone.0100252.s004.tif]
